# Genomic heterogeneity of multiple synchronous lung cancers in Chinese population

**DOI:** 10.1002/cam4.6928

**Published:** 2024-02-04

**Authors:** Lei Zhao, Jin Wang, Yixiang Zhang, Peng Wang, Changsheng Lv, Shilei Zhao, Tao Guo, Fengzhou Li, Chundong Gu, Yuntao Zhu

**Affiliations:** ^1^ Department of Thoracic Surgery the First Affiliated Hospital of Dalian Medical University Dalian China

**Keywords:** clonality index, genomic features, intrapulmonary metastasis, multiple synchronous lung cancers

## Abstract

**Introduction:**

It is clinically challenging to infer the phylogenetic relationship between different tumor lesions of patients with multiple synchronous lung cancers (MSLC), whether these lesions are the result of independently evolved tumor or intrapulmonary metastases.

**Methods:**

We used the Illumina X10 platform to sequence 128 stage I lung cancer samples collected from 64 patients with MSLC. All samples were analyzed for mutation spectra and phylogenetic inference.

**Results:**

We detected genetic aberrations within genes previously reported to be recurrently altered in lung adenocarcinoma including, EGFR, ERBB2, TP53, BRAF, and KRAS. Other putative driver mutations identified were enriched in RTK‐RAS signaling, TP53 signaling, and cell cycle. Also, we found some interesting cases, two cases that carried EGFR L858R and T790M co‐mutation in one tumor and another tumor with only EGFR 19del, and 1 case with two KRAS hotspots in the same tumor. Due to the short follow‐up time and early stage, further investigation is needed to determine whether this unique mutation profile will affect their progression‐free survival (PFS) and overall survival (OS). Regarding genetic evolution analysis among 64 tumor samples, 50 of them display distinct mutational profiles, suggesting these are independently evolved tumors, which is consistent with histopathological assessment. On the other hand, six patients were identified to be intrapulmonary metastasis as the mutations harbored in different lesions are clonally related.

**Conclusion:**

In summary, unlike intrapulmonary metastases, patients with MSLC harbor distinct genomic profiles in different tumor lesions, and we could distinguish MSLC from intrapulmonary metastases via clonality estimation.

## 1 INTRODUCTION

Lung cancer is the leading cause of global cancer mortality. In recent years, advancements in early detection and diagnostic techniques have led to an increased identification of nodules. Multiple primary lung cancers (MPLCs) refer to the occurrence of multiple primary tumors in different areas of the lungs within a single patient, which can be synchronous or metachronous.^1^ The prevalence of multiple synchronous lung cancers (MSLC) has been reported to range from 0.2% to 8% among lung cancer cases,^2^, ^3^, ^4^, ^5^ distinguishing them from pulmonary metastases. Treatment plans for MPLCs depend on whether they are MSLCs or metastatic lung cancers and their TNM staging.^6^

Currently, there is no universally accepted standard for diagnosing and treating MSLCs; however, most clinicians refer to the criteria published by Martini and Melamed in 1975.^1^ In 2003, the American College of Chest Physicians (ACCP) suggested that molecular markers could aid in distinguishing genomic features when tumor foci share the same histological subtype.^7^ Over time, these molecular markers have evolved from microsatellite analysis^8^ and loss of heterozygosity assessments to mutation status evaluations of gene's such as TP53^9^ and large‐scale gene mutations.^10^, ^11^, ^12^

Coincidentally, lots of studies have reported differences in specific cancer gene mutations and chromosome aberrations between MSLCs.^13^, ^14^, ^15^, ^16^ Ramsey Asmar et al. found concordance in oncogenic driver mutations (e.g., EGFR, KRAS) between primary tumors and metastasis^17^; conversely Yu Liu et al. highlighted genomic heterogeneity within MSLC cases.^13^ Snehal B. Patel et al.^18^ confirmed this view by 50 gene panel in 2017: they observed high mutational concordance between primary‐metastatic pairs with consistent driver mutations but distinct mutational characteristics among MSLC cases. Several similar studies support these findings.^11^, ^12^, ^19^, ^20^

However, the consistency of Histologic Prediction via CT imaging with genomic features remains controversial due to the lack of benchmarking against molecular approaches. A study by Chang et al. found that 22% (17 of 76) patients showed discordant results between prospective histologic prediction and final molecular classification. Furthermore, the discordance rate was significantly higher for intrapulmonary metastasis (IPMs) (11/25, 44%) than MSLCs (6/51, 12%).^21^ Till now, in clinical practice, gene markers have not been widely used in diagnosing MSLC due to the absence of reliable markers and algorisms to determine whether two or more tumor foci are of different origins. In this study, we analyzed 64 patients including 128 samples using NGS targeted gene sequencing to explore their mutation profiles and developed a clonality index (CI) through a mathematical algorithm to distinguish MSLC from intrapulmonary metastasis.

## 2 MATERIALS AND METHODS

### 2.1 Patients

This clinical trial was initiated in 2018, during which we sequenced a total of 128 stage I lung cancer samples collected from 64 patients with MSLCs using the Illumina X10 platform between October 2018 and October 2019. Resectable specimens were obtained for High Throughput Sequencing (HTS) along with matched blood normal controls. All patients were diagnosed with multiple synchronous lung adenocarcinomas by pathologists at the first visit. None of the patients had extrathoracic metastasis or received neoadjuvant therapies before surgery, and their clinical data, including age, sex, smoking history, tumor pathological information, and tumor biomarker information was collected. Each patient had only two lesions. Each patient had only two lesions that shared similar histologic type, radiographic appearance and growth patterns; thus they could not be diagnosed as either MSLC or intrapulmonary metastases.

This study was approved by the Local Ethics Committee at the First Affiliated Hospital of Dalian (YJ‐KY‐FB‐2019‐18), and written informed consent was obtained from the patient.

### 2.2 Sample collection and genomic profiling

Tumor tissues were snap‐frozen and stored in liquid nitrogen after collection; Tumor tissue samples were less than 1.5 cm × 1.5 cm in size with a thickness ranging from 2 to 5 mm. Genomic DNA was extracted from fresh‐frozen samples using QIAamp DNA Mini kit (Qiagen, 51306, Valencia, CA, USA) according to the manufacturer' s protocol, and fragmented for constructing a library using KAPA Hyper Prep kits (KAPA, KK8504) and captured using the Agilent SureSelect XT Human All Exon v5 kit (Agilent Technologies, Santa Clara, CA, USA). Using Genetron's 509 cancer panel, exons of major cancer genes of all tumor tissue and corresponding WBC were sequenced on Illumina NovaSeq6000 provided by Genetron Health.

### 2.3 Statistical analysis

Both tumor DNA samples and their matched normal DNA samples were subjected to standardized computational workflow for analysis: Raw sequence data (FastQ format) underwent trimming and filtering using Trimmomatic 0.33 using the following parameters:1.ILLUMINACLIP:TruSeq3‐PE‐adapter.fa:2:30:10:8:true; 2. TRAILING:3; 3. SLIDINGWINDOW:4:15; 4. MINLEN:36.^22^ Paired‐end clean reads then were aligned to the human reference genome (hg19) using the Burrows–Wheeler Aligner (BWA, version 0.7.10‐r789) with default parameters.^23^ Duplicate removal, local realignment, and base quality recalibration were performed using PICARD (http://broadinstitute.github.io/picard/, version 1.103) and the Genome Analysis Toolkit (GATK, version 3.1‐0‐g72492bb).^24^ Somatic single nucleotide variations (SNVs) were called using Mutect (version 3.1‐0‐g72492bb),^25^ incorporating the Catalog of Somatic Mutations in Cancer (COSMIC) v54 and dbSNP138 as reference sets of known somatic and germline mutations, respectively. Small indels were identified through strelka (version 1.0.14) employing defaults parameters with BAM as input.^26^ The effects of variants were annotated using Variant Effect Predictor (VEP). CREST was utilized for detection of somatic structural variants (SVs), while Control‐FREEC was employed for analysis of somatic copy‐number variants including loss of heterozygosity (LOH). The clonality index (CI) was calculated according to previous methods.^27^

For Calculation of CI, we leveraged genomic data of lung adenocarcinoma samples in TCGA to construct positive (IPM) and negative (MPLC) dataset. In the positive dataset, we randomly selected a proportion of somatic mutations from each lung adenocarcinoma sample twice (with replacement) to generate pairs of mutation sets. For each shared mutation, we modeled the probability of observing it in both samples as binomial distribution $P\left(X\right)=C_{n}^{k}p^{k}{\left(1-p\right)}^{n-k}$, where *p* is the population prevalence of such mutation in lung adenocarcinoma cohort in TCGA. Therefore, the probability of observing a given set of M identical mutations in the two samples is given by $\prod_{m=1}^{M}P{\left(X\right)}_{m}$. The CI is defined as $\mathrm{CI}=-\log_{10}\prod_{m=1}^{M}P{\left(X\right)}_{m}$. In contrast, for the negative dataset, pairs of mutation sets were generated from different samples instead of the same sample. The CI was subsequently calculated through same procedure. CIs obtained from positive to negative datasets represent the clonality of IPM and MPLC respectively. Optimum CI cut‐off was then determined according to AUROC curve analysis.

To objectively establish a threshold for clonal relatedness, we utilized the mutational data from 514 unrelated Lung adenocarcinoma from TCGA. As a positive control (i.e., clonally related) to simulate heterogeneity between biologically related samples, we randomly selected 40%, 60%, and 80% of the set of mutations from the pool of 505 unrelated samples. Conversely, as a negative control (i.e., unrelated), we randomly selected an equivalent number of pairs (i.e., 3 × 505 = 1515) of unrelated samples from TCGA.

The R package “ROCR” was utilized to optimize accuracy and determine the optimal cut‐offs. To mitigate over‐fitting, this process was repeated 100 times to establish the median and 95% confidence interval of the optimum cut‐off. For the targeted panel of 509 genes, the median optimum cut‐off was 7.77 (95% confidence interval 7.63–7.97), and with the optimum cut‐offs, the median accuracy was 96.16% (95% confidence interval 96.03%–96.17%), the median sensitivity was 93.12% (95% confidence interval 92.9%–93.16%), and the median specificity 99.44% (95% confidence interval 99.26%–99.45%).

## 3 RESULTS

### 3.1 Patient

A total of 64 patients at the First Affiliated Hospital of Dalian Medical University between July 2018 and April 2020 were identified based on multiple primary lung cancer by clinical‐pathologic criteria. The clinical information of these patients is summarized in Table 1 and Table S1. All patients presented with two histologically distinct tumor lesions. The median age was 56 years (ranging from 29 to 75 years), 53 of them were non‐smokers. Among all these patients, 27 (42%) patients had both tumors in the same lobe, 37 (58%) in different lobes. For the 128 tumors, all of them were diagnosed as adenocarcinoma. None of the enrolled patients received neoadjuvant chemotherapy or any other anticancer therapy prior to surgery.

**TABLE 1:** Clinical characteristics of 64 patients.

Patients' characteristics	Value (%)
Total patients	64
Sex	
Female	45 (70%)
Male	19 (30%)
Age at first diagnosis	
Median	56
Range	29–75
Smoking history	
No	53 (83%)
Yes	11 (17%)
Stage	
IA	54 (84%)
IB	10 (16%)
Location	
RUL	45
RML	13
RLL	22
LUL	31
LLL	17

Abbreviations: LLL, left lower lobe; LUL, left upper lobe; RLL, right lower lobe; RML, right middle lobe; RUL, right upper lobe.

### 3.2 The genetic landscape of MSLC

**FIGURE 1:**
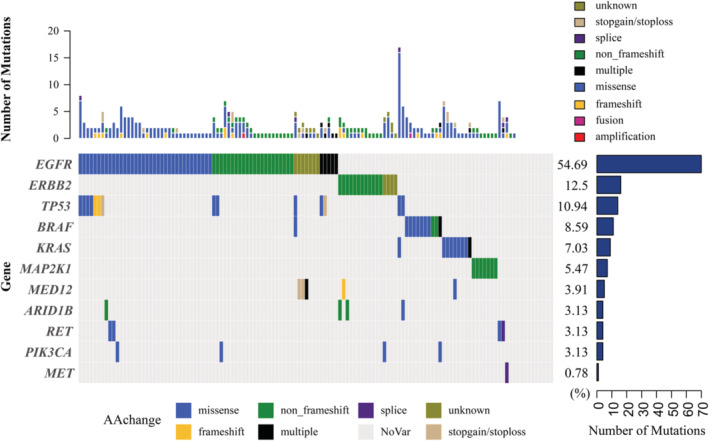
The mutation landscape of 128 samples.

We analyzed the mutation pattern of 128 samples and identified a total of 292 genetic alterations, including 290 somatic, non‐synonymous mutations, one amplification and one fusion. The average number of mutations per tumor was 0.95 (ranging from 0 to 15.08). Mutations were detected in at least one tumor in 118 tumors (92.2%), while no mutation was detected in the remaining 10 tumors (7.8%). The mutational landscape is shown in Figure 1. Recurrently mutated genes included EGFR (*n* = 70), ERBB2 (*n* = 15), TP53 (*n* = 14), BRAF (*n* = 10), and KRAS (*n* = 9). ALK, RET and ROS1 fusion were not found in these tumors, MET exon 14 skipping was detected in one sample. Among the tumors with EGFR mutation (*n* = 70), L858R mutation was present in 38 (54.3%) tumors, with two samples also harboring EGFR T790M mutation. Two samples carried both G719A (exon 18) and L861Q (exon 21) mutations and one sample carried only G719A (exon 18) mutation. Exon 19 deletions were detected in 23 (32.9%) tumors, whereas six additional samples presented exon 20 insertion (Table S1). KRAS mutations were found in nine samples (9 were G12 mutation, 1 was Q61H), with one (Case 31) carrying both G12D and G12V mutations. Also, all of the KRAS mutations were not associated with EGFR mutations. In the 11 samples which had BRAF mutation, two of them were BRAF V600E mutation. None of the tumors had co‐drivers of EGFR, KRAS, BRAF, ERBB2 and MET. TMB was calculated and the median TMB was 0.79 (from 0 to 15.08).

### 3.3 Genomic heterogeneity of multiple synchronous lung cancers

**FIGURE 2:**
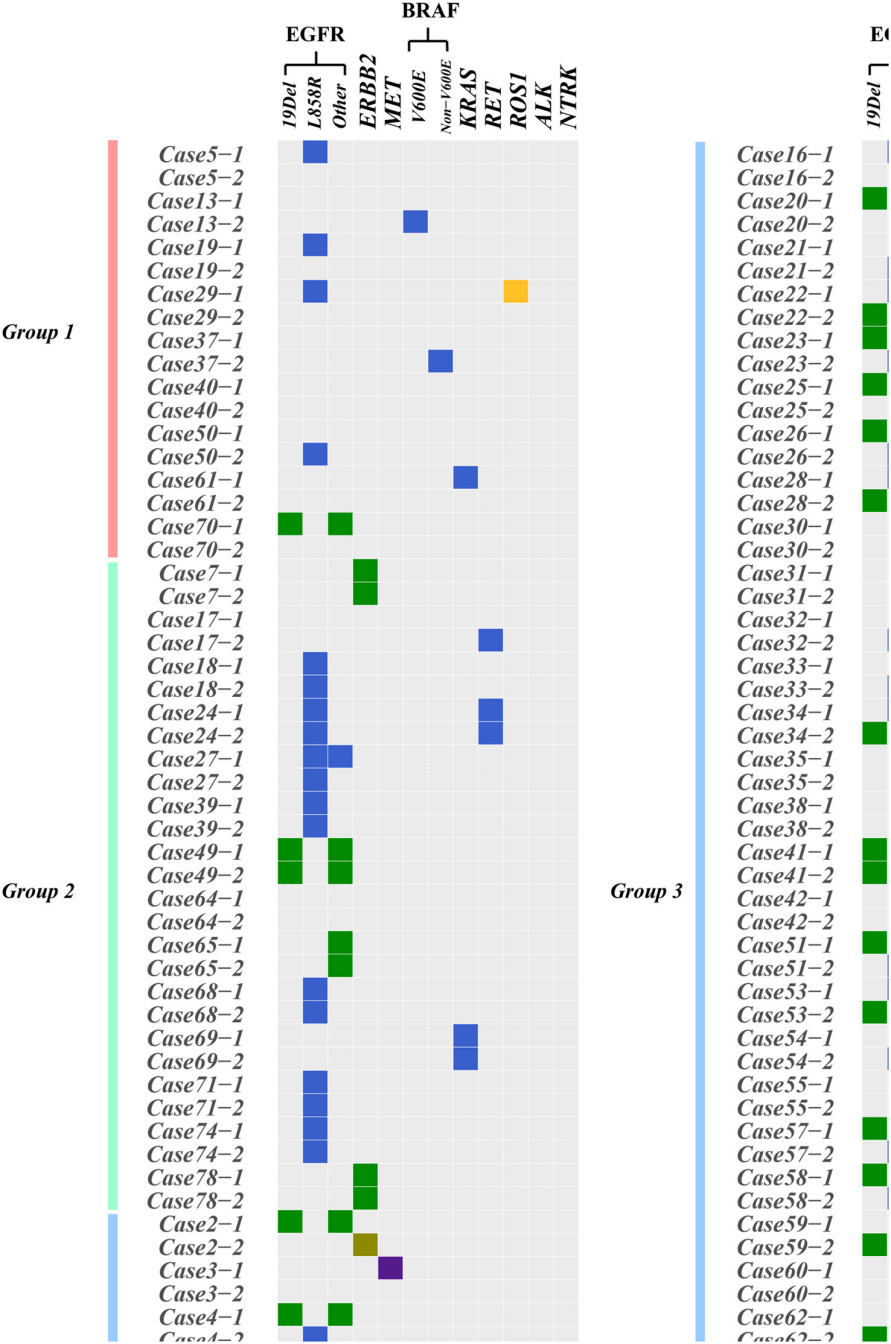
Somatic mutations spectra in different groups of MSLC.

The driver mutation profiles of different tumor foci in the same individuals were compared to each other. Based on whether it contains the same driver mutation among different tumors in the same individual, 64 patients were separated into 3 groups. Group 1 comprised 10 patients who had one tumor without any detected mutations. Group 2 included individuals who either had identical driver mutations or no driver mutation but exhibited other gene mutations that were shared between different tumors. Samples in group 3 displayed distinct driver mutations or no other shared gene mutations within an individual. There were 10 (15.6%), 14 (21.9%), and 40 (62.5%) patients in group 1, 2, and 3 separately (Figure 2). In group 1 and 3, for there were no same mutations in the two separated tumors, there was concordance with the conclusions drawn from pathology and imaging evidence; however, for individuals in group 2 who carried either the same driver mutation or other shared mutations (without a driver mutation), further investigation is required to determine their origin.

### 3.4 Define clonality index to classify MSLC with intrapulmonary metastasis

For the 14 patients in group 2, which shared gene mutation between different tumors in one individual, we defined a clonality index (CI) to differentiate MSLC with intrapulmonary metastasis. This index assesses the likelihood of co‐occurring mutations beyond chance, considering all somatic synonymous and non‐synonymous alterations. The result of these 14 patients and the cut‐off for CI is shown in Table 2. Among them, six patients have CI dramatically above the cut‐off which means these tumors might be intrapulmonary metastasis. In addition to common driver mutations, these individuals also exhibited shared passenger mutations across their tumors. The remaining eight patients had CI close to the cut‐off threshold (7.77, Table 2), necessitating further confirmation through integration of additional clinical information. Notably, the shared mutations observed in these cases were limited to hotspot regions within driver gene.

**TABLE 2:** The clonality index of 14 individuals.

Case	Driver gene alterations	Clonality index (CI)	Final class
7	ERBB2 (Tyr772_Ala775dup)	19.86	IPM
17	None	31.93	IPM
18	EGFR (L858R)	7.77	MSLC
24	EGFR (L858R)	29.76	IPM
27	EGFR (L858R)	7.77	MSLC
39	EGFR (L858R)	7.77	MSLC
49	EGFR (p.Glu746_Ala750del)	8.37	MSLC
64	None	10.45	IPM
65	EGFR (p.Ala767_Val769dup)	10.45	IPM
68	EGFR (L858R)	7.77	MSLC
69	KRAS (p.G12C)	6.69	MSLC
71	EGFR (L858R)	7.77	MSLC
74	EGFR (L858R)	7.77	MSLC
78	ERBB2 (Tyr772_Ala775dup)	9.06	IPM

*Note*: Cut‐off is 7.77.

Abbreviations: ADC: adenocarcinoma; IPM, intrapulmonary metastasis; MSLC, multiple synchronous lung cancers.

## 4 DISCUSSION

In recent years, there has been an increasing incidence of multiple synchronous lung cancer (MSLC) cases being diagnosed. MSLC generally has a better prognosis. However, distinguishing it from metastatic lung cancer remains a challenge in clinical practice. Many clinical cases cannot be solely diagnosed based on tumor location and histological features alone. With the recent advancements in genomic technology, we aimed to characterize the genomic features of individual tumor foci in MSLC and explore the potential use of genomic markers to facilitate its diagnosis.

There have been several reports suggesting the heterogeneity of different tumor foci in MSLC.^11^, ^12^, ^28^, ^29^ Yu Liu et al. conducted a genomic analysis of 15 lung adenocarcinomas and one regional lymph node metastasis from 6 patients, revealing distinct genomic profiles in all 15 lung tumors.^11^ Another study utilized a combined histo‐molecular algorithm based on 22 hotspot genes to classify MSLC with intrapulmonary metastasis and guide adjuvant treatment decisions.^30^ A recent study performed targeted sequencing of a 464‐gene panel in 16 patients with multiple tumors, demonstrating the utility of DNA sequencing when pathology is inadequate to make a conclusive diagnosis.^31^ However, these studies had limited sample sizes or focused on fewer genes. In our study, we employed NGS sequencing to investigate the 509 genes across different tumors from 64 patients with multiple synchronous lung cancer at initial diagnosis. Our genomic study suggested that in 50 patients, different tumor foci do not have shared mutations, suggesting the tumor foci are of independent origin and are likely synchronous multiple primary lung cancers. The genotyping results align with histological and imaging‐based conclusions. From the perspective of evolutionary biology, tumorigenesis follows Darwin's theory of natural selection, choosing a suitable way in both expansions and constraints. The continual appearance of random mutagenesis and universal selection of positive and negative induce the distinct tumorigenic alterations of each lesion. Sequencing technologies can effectively identify the clonality among multiple lesions within the same patient affected by certain genetic changes to distinguish the primary lesions from the metastatic ones. As evidenced by previous studies, the molecular spectrum of independent clones exhibits significant heterogeneity, and lesions sharing a high degree of great similarity in the mutation spectrum tend to be IMs.

**FIGURE 3:**
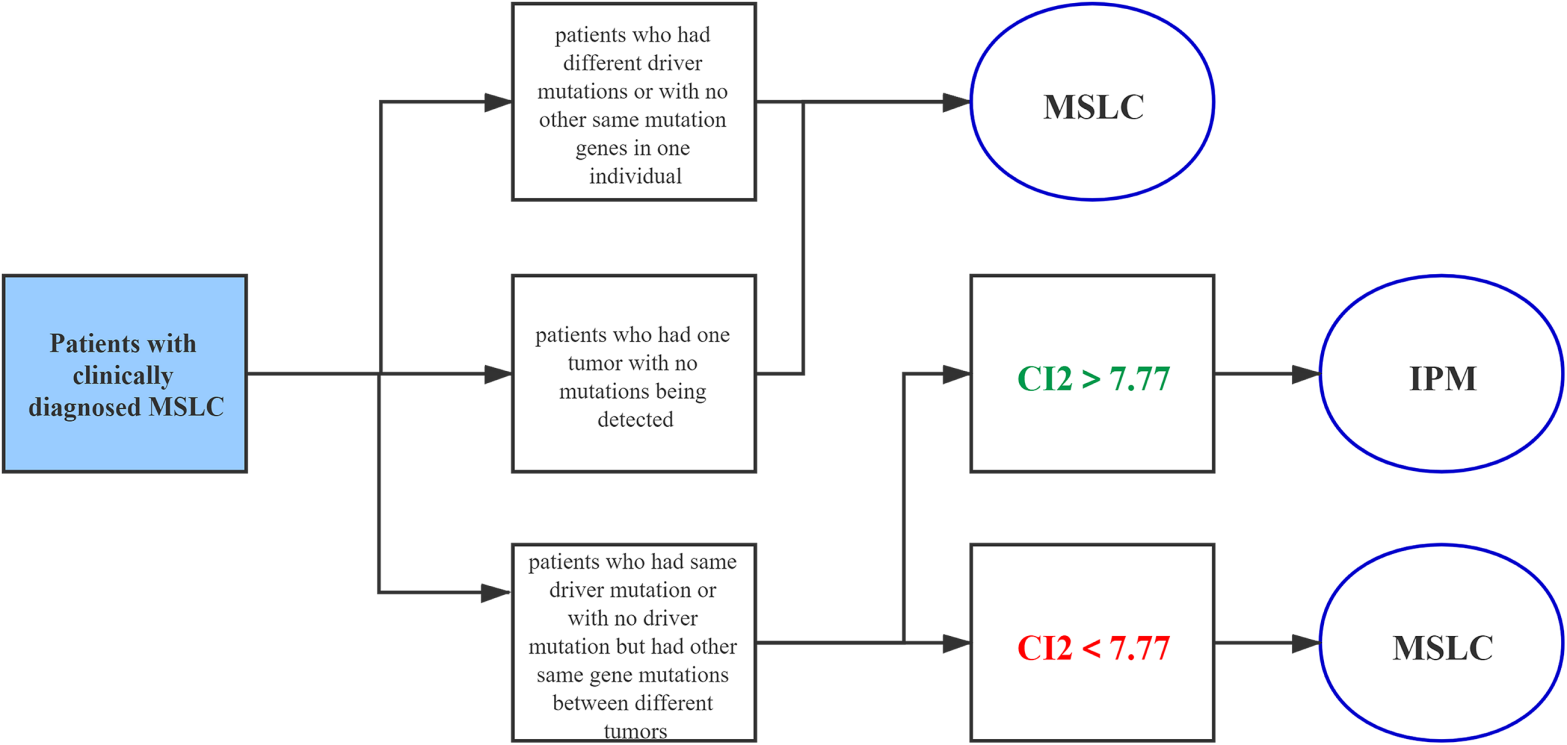
Flowchart for classification of MSLC using next‐generation sequencing.

To distinguish MPLC and IPM, the Clonality index (CI) is calculated to determine whether the likelihood of two samples derived from individual patient sharing the same mutations beyond expected co‐occurrence by chance. Six patients exhibited a significantly higher CI above the cut‐off, indicating that the foci are likely metastasis. Eight patients had CI close to cut‐off, suggesting that although they may have originated from different sources initially, they evolved in a similar direction under comparable selection pressure. Based on our data, we propose a flow diagram for classification of MSLC using next‐generation sequencing and CI2 (Figure 3). Overall, retrospective histologic reassessment of cases unambiguously classified by NGS will provide an unprecedented benchmark against which to refine histologic criteria for distinguishing MSLC and IPMs in future studies.

Additionally, there were some interesting cases in the study. We found 2 cases (Case 73 and Case 57) had primary EGFR T790M mutation. According to the previous report, which focused on stage III–IV patients, the results demonstrated that de novo T790M mutation was more likely to carry concurrent EGFR L858R mutation,^32^, ^33^ resulting in a favorable response to osimertinib treatment. In our cohort, the only 2 cases harboring primary EGFR T790M mutation were co‐mutated with EGFR L858R, indicating simultaneous occurrence of these two mutations at similar frequencies. Furthermore, a meta‐analysis encompassing data from 22 clinical trials and reported that de novo EGFR T790M was an unfavorable predictive and prognostic marker in the late stage lung cancer.^34^ However, there was no research focus on stage I lung cancer, and these early stage patients will not receive any treatment after surgery, whether the primary EGFR T790M will be an unfavorable prognostic marker need further investigation. Another unique case (Case 31) is a 56 years woman, one of the tumor carried two activation site (G12D and G12V), and the mutation frequency was 5.7% and 7.8%, respectively. To the best of our knowledge, there are no existing studies reporting such phenomena; therefore follow‐up results are necessary to ascertain whether this tumor exhibits increased aggressiveness.

Our study still had some deficiencies, for it was a single center study and the follow‐up data was not available at this time. The sample size needs to be increased and intrapulmonary metastasis data would better be included. For the application of CI, we constructed positive and negative datasets using genomic data in TCGA which were dominated by Western individuals. The difference in population prevalence of some driver mutations between western population and eastern population may introduce bias into the model and cause inaccuracy of CI calculation when applied to Chinese population. Furthermore, to our knowledge, CI was only used in gynecological tumors like Synchronous Endometrioid Endometrial and Ovarian Carcinomas. Therefore, we should accumulate more data for lung cancer to optimize the application of CI.

Our results showed that the conclusions drawn from genotyping results are highly consistent with that drew from histology and imaging information, indicating that genomic markers can serve as valuable tools for facilitating clinical diagnosis. With the rapid development of genomic and sequencing technologies in recent years, the use of genomic markers has become increasingly prevalent in diagnostic settings. In particular, these markers can be useful in diagnosis and guide treatment plans for many diseases such as breast cancer. Our study here provides evidence that the incorporating genome profiling in addition to traditional histological and imaging approaches can provide more comprehensive analyses for the diagnosis of MSLC.

## AUTHOR CONTRIBUTIONS

**Lei Zhao:** Conceptualization (supporting); data curation (lead); writing – review and editing (lead). **Jin Wang:** Data curation (equal). **Yixiang Zhang:** Data curation (supporting). **Peng Wang:** Data curation (supporting). **Changsheng Lv:** Formal analysis (supporting). **Shilei Zhao:** Formal analysis (supporting). **Tao Guo:** Formal analysis (supporting). **Fengzhou Li:** Formal analysis (supporting); writing – original draft (lead). **Chundong Gu:** Conceptualization (equal); supervision (equal); writing – review and editing (equal). **Yuntao Zhu:** Conceptualization (equal); supervision (equal); writing – review and editing (equal).

## FUNDING INFORMATION

This work was supported by the National Natural Science Foundation of China (No: 81774078).

## CONFLICT OF INTEREST STATEMENT

All the authors declare that they have no conflict of interest.

## ETHICS STATEMENT

All procedures conducted in studies involving human participants were in accordance with the ethical standards of the institutional and/or national research committee and with the 1964 Helsinki Declaration and its later amendments or comparable ethical standards.

## Supporting information


Table S1.

## Data Availability

The datasets used and/or analyzed during the current study are available from the corresponding author on reasonable request.
